# Causal relationship between genetic proxies for calcium channel blockers and the risk of depression: a drug-target Mendelian randomization study

**DOI:** 10.3389/fpsyt.2024.1377705

**Published:** 2024-05-10

**Authors:** Chaoyi Ye, Tingjun Wang, Huajun Wang, Guili Lian, Liangdi Xie

**Affiliations:** ^1^ Department of Geriatrics, The First Affiliated Hospital of Fujian Medical University, Fuzhou, China; ^2^ Fujian Hypertension Research Institute, The First Affiliated Hospital of Fujian Medical University, Fuzhou, China; ^3^ Department of Cardiology, Xiamen Cardiovascular Hospital of Xiamen University, School of Medicine, Xiamen University, Xiamen, China; ^4^ Branch of National Clinical Research Center for Aging and Medicine, Fujian Province, The First Affiliated Hospital of Fujian Medical University, Fuzhou, China; ^5^ Fujian Provincial Clinical Research Center for Geriatric Hypertension Disease, The First Affiliated Hospital of Fujian Medical University, Fuzhou, China; ^6^ Department of Geriatrics, National Regional Medical Center, Binhai Campus of the First Affiliated Hospital, Fujian Medical University, Fuzhou, China

**Keywords:** depression, calcium channel blockers, hypertension, mendelian randomization analysis, genome-wide association study

## Abstract

**Background:**

Calcium channel blockers (CCBs) are widely used in the clinical management of hypertension. Depression, a common comorbidity of hypertension, is an important issue in the management of hypertension. However, the impact of CCBs on depression risk remains controversial. We aim to investigate the causal effect of CCBs on depression through drug-target Mendelian randomization (MR) analysis.

**Methods:**

To proxy CCBs, we utilized the genetic variations located in or around drug target genes that were related to systolic blood pressure (SBP). Coronary artery disease (CAD) served as the positive control outcome. Genetic summary data of SBP, CAD, and depression were obtained from genome-wide association studies (GWAS) based on European population. Inverse variance weighted (IVW) method was applied as the main analysis to estimate the causal effect. Cochran’s Q test, MR-Egger intercept, MR pleiotropy residual sum and outlier (MR-PRESSO) and leave-one-out sensitivity analysis were used to test the robustness of the results. Meta-analysis was applied to further confirm whether causal relationships existed between CCBs and depression.

**Results:**

The IVW results failed to reveal any causal relationship between genetic proxies for CCBs and depression (*P* > 0.05). Cochran’s Q test showed no evidence of heterogeneity (*P* > 0.05). The MR-Egger intercept test suggested no evidence of directional pleiotropy, and the MR pleiotropy residual sum and outlier (MR-PRESSO) global test for horizontal pleiotropy was also not significant (*P* > 0.05). Leave-one-out analysis did not reveal any genetic variant that influenced the results. In addition, the meta-analysis further confirmed the absence of a causal relationship.

**Conclusion:**

The present study indicates no association of genetic proxies for CCBs with depression. Further studies are necessary to provide definitive evidence.

## Introduction

Hypertension, a worldwide chronic disease, is a major cardiovascular risk factor that affects a large number of adults (1). Depression is a frequent comorbidity among patients with hypertension (2). A systematic review showed that approximately 27% of patients with hypertension experience depressive symptoms (3). Major depressive disorder, current depressive symptoms and a history of depression have all been linked to an increased risk of cardiovascular morbidity and mortality (4, 5). Therefore, in patients with hypertension and cardiovascular diseases (CVD), preventing the development of depression is very important.

Calcium channel blockers (CCBs) are recommended as part of an antihypertensive treatment that acts by preventing the influx of extracellular calcium to achieve vasodilation (6). Previous studies have shown associations between the use of CCBs and depression (7–11). However, the current evidence is not adequate partly due to inconsistencies among the existing studies. For example, several epidemiological studies have shown that the administration of CCBs was associated with a high risk of depressive symptoms (7, 8). The results from some meta-analyses also implied that risk of depression was higher among individuals on the treatment of CCBs (9, 10). However, Kessing et al. reported that the consistent use of CCBs was associated with a lower rate of depression (11). The differences in ethnicities, outcome definitions, and bias caused by possible confounding factors may explain these inconsistent results. In general, observational epidemiological studies are not sufficient to establish causation. Although randomized clinical trials (RCTs) are optimal for determining causality, they have restrictions regarding resource availability, cost, and ethics (12).

With the rapid advance of genetics, Mendelian randomization (MR) has been widely used to study the mechanism of human disease in recent years. MR is an approach that uses genetic variants as instrumental variables for investigating causal associations from observational data (13). As alleles are randomly assigned at conception and are often independent to environmental or lifestyle factors, MR is analogous to a natural RCT (14). Given the greater open access of genome-wide association studies (GWAS), the causal relationship between traits and diseases could be evaluated efficiently by MR. Drug-target MR utilizes genetic variations in or around a drug target-encoding gene to obtain a causal estimate of the protein effect on multiple outcomes (15). In this study, we performed a drug-target MR analysis to examine the causal association of CCBs with depression. We further conducted meta-analysis of multiple database results to ensure the data reliability.

## Materials and methods

### Ethics statement

The GWAS datasets used in this study are publicly available online (https://gwas.mrcieu.ac.uk/). Each GWAS included in this study obtained written informed consent from all participants and was approved by their respective local ethics board.

### Study design

**Figure 1:**
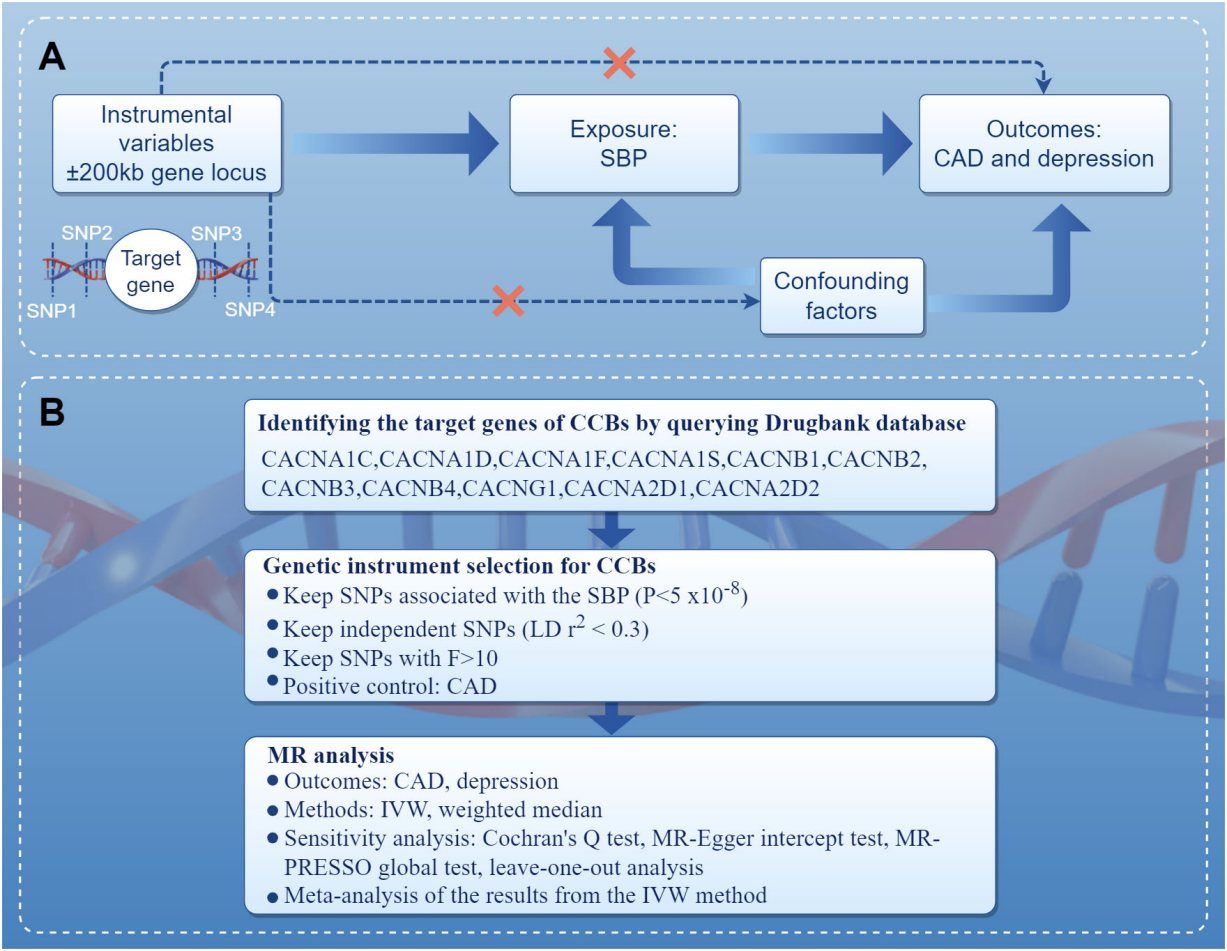
Diagram of the study design. **(A)** The drug-target MR framework in this study. To verify the existence of a causal relationship, the following conditions are necessary: (1) the instrumental variables are not associated with confounding factors (dashed line), (2) the instrumental variables must be associated with the exposure factor (solid line), and (3) the instrumental variables are not directly related to the outcome (dashed line). **(B)** The overall study design and workflow. CAD, coronary artery disease; CCBs, calcium channel blockers; IVW, inverse variance weighted; MR, Mendelian randomization; MR-PRESSO, Mendelian randomization pleiotropy residual sum and outlier; SBP, systolic blood pressure; SNPs, single nucleotide polymorphisms.

The present study performed MR in the European population to investigate the association of genetic proxies for CCBs with the risk of depression. The overall study design is shown in **Figure 1**. Genetic variations related to blood pressure lowering were identified in drug target genes as proxies for CCBs. For the causal estimates of MR analysis to be effective, three important assumptions must be satisfied (16). Firstly, genetic instruments should be strongly associated with the exposure. Secondly, genetic instruments should not be correlated with any confounders. Thirdly, genetic instruments should not be independently associated with outcome and should exclusively mediate the effects via exposure. A completed Strengthening the Reporting of Observational Studies in Epidemiology Using Mendelian Randomization (STROBE-MR) statement was provided (**Supplementary Table S1**) (17).

### Genetic instruments for proxy CCBs

The summary statistics for systolic blood pressure (SBP) were obtained from the MRC IEU Open GWAS database (https://gwas.mrcieu.ac.uk/) and GWAS Catalogue (https://www.ebi.ac.uk/gwas/). All GWAS were conducted in individuals of European ancestry (18–20). The information regarding the GWAS data were summarized in **Table 1**. Target genes of CCBs were identified based on the DrugBank database, including CACNA1C, CACNA1D, CACNA1F, CACNA1S, CACNB1, CACNB2, CACNB3, CACNB4, CACNG1, CACNA2D1, and CACNA2D2 (21). It is possible to simulate the effects of CCBs by obtaining instrumental variables that can target these genes to reduce SBP. The instrumental variables select single nucleotide polymorphisms (SNPs) that are located 200 kb upstream of the start and 200 kb downstream of the end of the target genes and are related to SBP. To eliminate the influence of strong linkage disequilibrium (LD) on the results, LD thresholds of r^2^ < 0.3 and *P*<5×10^-8^ were used to choose the significant and independent SNPs. One of the assumptions of MR is that the SNPs do not directly influence the outcome. Therefore, the PhenoScanner website (http://www.phenoscanner.medschl.cam.ac.uk/) was explored to examine whether the enrolled SNPs were directly related to the outcome. The genetic instrument strength was evaluated by the proportion of explained variation (R^2^) and F statistics. We calculated the R^2^ and F statistics using the following formula: R^2 = ^2 × MAF × (1 − MAF) × β^2^, F=[(N-K-1)/K] × [R^2^/(1-R^2^)], where MAF is the minor allele frequency, β is the effect value of the genetic variant in the exposure, N is the sample size of the exposure GWAS, and K is the number of genetic instruments employed (22). To satisfy the strong association with exposure, we selected SNPs with F statistics greater than 10 as genetic instruments to avoid weak instrument bias (23). The positions of target genes and the corresponding SNP numbers are presented in **Supplementary Table S2**. Details of the selected SNPs and genes investigated are presented in **Supplementary Tables S3–S6**. If at least 3 SNPs that fulfilled the above filtering criterion, subsequent statistical analyses will be conducted.

**Table 1:** Summary of the GWAS included in this study.

Variables	Data codes	Source of sample ethnicity	Sample size	Author	Year of publication	PMID
Systolic blood pressure	ieu-b-38	European	757,601	Evangelou, E	2018	30224653
Systolic blood pressure	ukb-b-20175	European	436,419	Ben Elsworth	2018	NA
Systolic blood pressure	ebi-a-GCST90029011	European	469,767	Loh PR	2018	29892013
Systolic blood pressure	ebi-a-GCST90018972	European	340,159	Sakaue S	2021	34594039
Coronary artery disease	ebi-a-GCST005195	European	547,261	van der Harst P	2017	29212778
Major depression	ieu-b-102	European	500,199	Howard DM	2019	30718901

Datasets with relatively large sample sizes were selected in this study.

### Source of outcomes

Genetic instruments for major depression were extracted from a recent GWAS meta-analysis that included UK Biobank and PGC data. The study included 500,199 individuals of European ancestry, including 170,756 patients and 329,443 control individuals (**Table 1**) (24). In UK Biobank data, depression comprise a diverse group of diagnoses: “broad depression” denoting self-reported help-seeking behavior for mental health difficulties (e.g., nerves, anxiety, tension, or depression); “probable major depressive disorder” marked by self-reported depressive symptoms and depressive related impairment; and major depressive disorder identified from hospital admission records (24). In the PGC cohort, depression comprises a set of phenotypes, obtained through structured interviews and wider criteria (24). Depression was diagnosed based on internationally recognized criteria (DSM-IV, ICD-9, or ICD-10). Coronary artery disease (CAD) was used as a positive control outcome given that CCBs have been shown to have cardiovascular protective effects. CAD GWAS summary data were obtained from the CARDIoGRAMplusC4D consortium and UK Biobank, which included 122,733 patients and 424,528 control individuals (**Table 1**) (25).

### Data analysis

There are many reports regarding the prevention of cardiovascular events by CCBs in patients with CAD (26). Thus, we used the GWAS summary statistics on CAD as the positive outcome control to examine the effectiveness of instrumental variables. The exposure-related drug targeting instrumental variables were harmonized with the outcome datasets, and then inverse variance weighted (IVW) and weighted median were used for analysis. The IVW was used as the main MR analysis because it provides the most precise effect estimates, and most of the publications used it as the main analysis (27). By performing the Wald ratio estimates of individual SNPs, the IVW method combines them into one cumulative causal estimate (28). To verify the robustness of the MR results, heterogeneity and pleiotropy tests were performed. The heterogeneity among the SNPs was evaluated by Cochran’s Q test based upon IVW. In detail, no heterogeneity was detected if the *P* value of Cochran’s Q test was >0.05 (29). The MR-Egger intercept and Mendelian randomization pleiotropy residual sum and outlier (MR-PRESSO) global test were used to check pleiotropy, and *P* > 0.05 was considered to indicate that there was no evidence of pleiotropy (16, 30). MR-Egger could test for the presence of horizontal pleiotropy and account for horizontal pleiotropy using the MR-Egger intercept test. If there was no pleiotropy in the SNPs, the MR-Egger intercept decreased toward zero as the sample size increased (16). By detecting outliers among the included SNPs that are involved in the MR estimate, MR-PRESSO could evaluate horizontal pleiotropy (30). To evaluate the influence of each SNP on the analysis, we used the leave-one-out approach to remove each SNP individually and compared the IVW results with all variants. Finally, meta-analysis of estimates from the IVW was performed was performed on all data to enhance persuasion of our findings. All statistical analyses were performed using the “TwoSampleMR”, “ggplot2” and “meta” packages in R software (version 4.3.0).

## Results

### Positive control analysis

**Figure 2:**
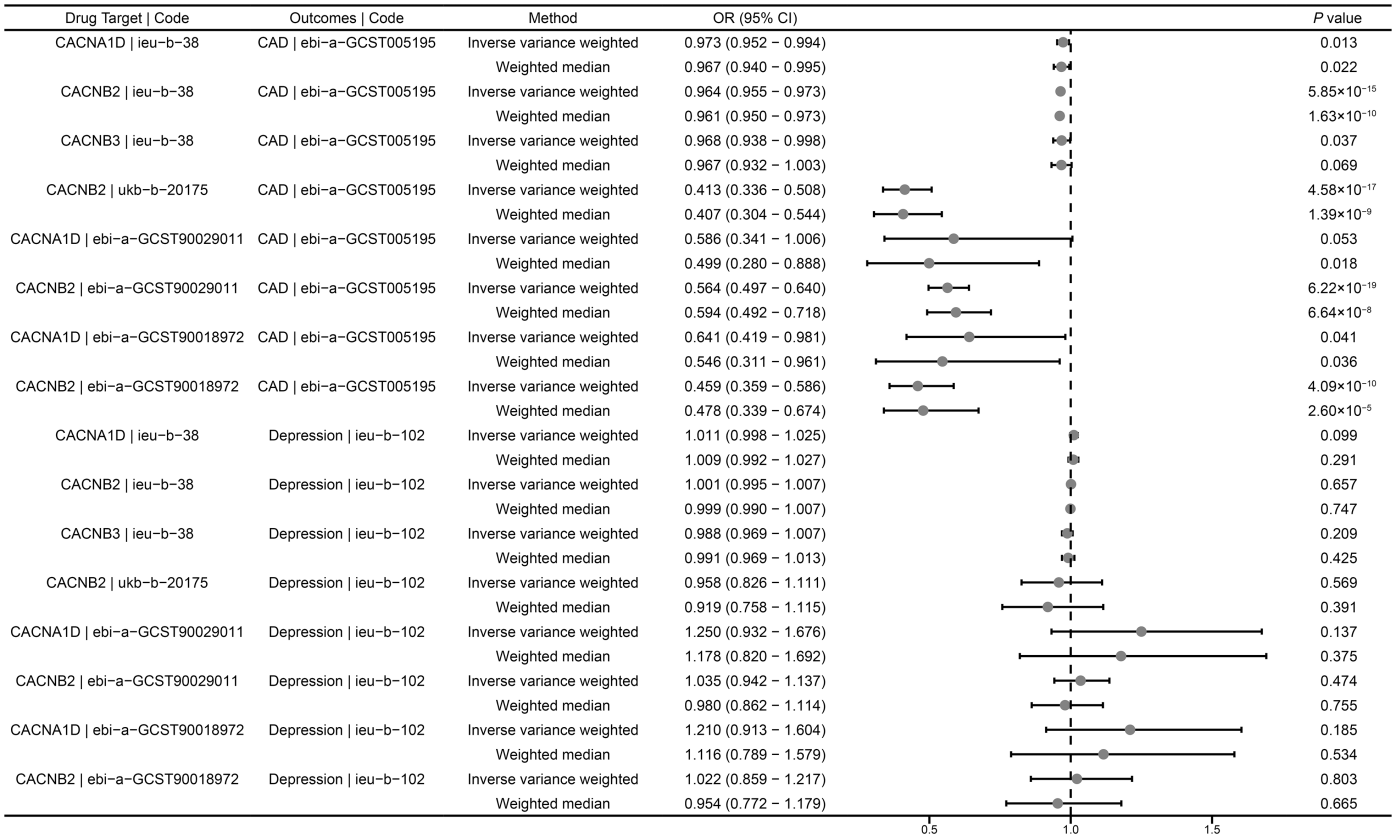
Summary results of drug-target MR. CAD, coronary artery disease; OR, odds ratio; CI, confidence interval.

In the positive control analysis, we observed significant associations between genetically proxied drug targets and lower risk of CAD, indicating the genetic instruments with good validity, except for genetically proxied inhibition of CACNA1D (ebi-a-GCST90029011), which showed a tendency towards protection but without statistical significance (OR = 0.586, 95% CI: 0.341-1.006, *P* = 0.053) (**Figure 2**). These findings further confirm the effectiveness of the genetic instruments.

### Drug-target MR analysis for depression

We investigated the causal role of CCBs in the risk of depression using a drug-target MR analysis. The results suggested that there was no causal relationship between genetically proxied inhibition of CACNB2 and the risk of depression (ieu-b-38: OR = 1.001, 95% CI: 0.995-1.007, *P* = 0.657; ukb-b-20175: OR = 0.958, 95% CI: 0.826- 1.111, *P* = 0.569; ebi-a-GCST90029011: OR = 1.035, 95% CI: 0.942-1.137, *P* = 0.474; ebi-a-GCST90018972: OR = 1.022, 95% CI: 1.217-0.859, *P* = 0.803) (**Figure 2**). Similarly, neither CACNA1D inhibition (ieu-b-38: OR = 1.011, 95% CI: 0.998-1.025, *P* = 0.099; ebi-a-GCST90029011: OR = 1.250, 95% CI: 0.932-1.676, *P* = 0.137; ebi-a-GCST90018972: OR = 1.210, 95% CI: 0.913-1.604, *P* = 0.185) nor CACNB3 inhibition (ieu-b-102: OR = 0.988, 95% CI: 0.969-1.007, *P* = 0.209) was significantly associated with the risk of depression (**Figure 2**).

### Sensitivity analysis

Heterogeneity was evaluated using Cochrane’s Q statistics. The results showed that there was no significant heterogeneity under the IVW model in any outcome (*P* > 0.05) (**Table 2**). The MR-Egger intercept and MR-PRESSO global test were also utilized to detect directional and horizontal pleiotropy, and all *P* values were higher than 0.05, indicating no evidence of pleiotropy (**Table 2**). No outliers were found by the MR-PRESSO test. To ensure the robustness of the results, leave-one-out analysis was applied by removing one SNP at a time to check whether individual SNPs influenced the results. It was shown that no single SNP had a significant influence on the overall estimates for CAD (**Supplementary Figure S1**) and depression (**Supplementary Figure S2**).

**Table 2:** Evaluation of heterogeneity and pleiotropy using different methods.

Drug Target | Code	Outcomes | Code	Heterogeneity test Cochran’s Q *P* value	Pleiotropy test	
			MR-Egger intercept *P* value	MR-PRESSO global test *P* value
CACNA1D | ieu-b-38	CAD | ebi-a-GCST005195	0.376	0.355	0.398
CACNB2 | ieu-b-38	CAD | ebi-a-GCST005195	0.097	0.402	0.133
CACNB3 | ieu-b-38	CAD | ebi-a-GCST005195	0.434	0.574	0.530
CACNB2 | ukb-b-20175	CAD | ebi-a-GCST005195	0.653	0.396	0.736
CACNA1D | ebi-a-GCST90029011	CAD | ebi-a-GCST005195	0.270	0.352	0.347
CACNB2 | ebi-a-GCST90029011	CAD | ebi-a-GCST005195	0.366	0.178	0.431
CACNA1D | ebi-a-GCST90018972	CAD | ebi-a-GCST005195	0.324	0.631	0.341
CACNB2 | ebi-a-GCST90018972	CAD | ebi-a-GCST005195	0.190	0.939	0.209
CACNA1D | ieu-b-38	Depression | ieu-b-102	0.656	0.338	0.686
CACNB2 | ieu-b-38	Depression | ieu-b-102	0.077	0.999	0.085
CACNB3 | ieu-b-38	Depression | ieu-b-102	0.714	0.404	0.759
CACNB2 | ukb-b-20175	Depression | ieu-b-102	0.204	0.357	0.225
CACNA1D | ebi-a-GCST90029011	Depression | ieu-b-102	0.609	0.423	0.675
CACNB2 | ebi-a-GCST90029011	Depression | ieu-b-102	0.070	0.999	0.072
CACNA1D | ebi-a-GCST90018972	Depression | ieu-b-102	0.324	0.631	0.341
CACNB2 | ebi-a-GCST90018972	Depression | ieu-b-102	0.190	0.939	0.209

CAD, coronary artery disease; MR-PRESSO, mendelian randomization pleiotropy residual sum and outlier.

### Meta−analysis of IVW methods

**Figure 3:**
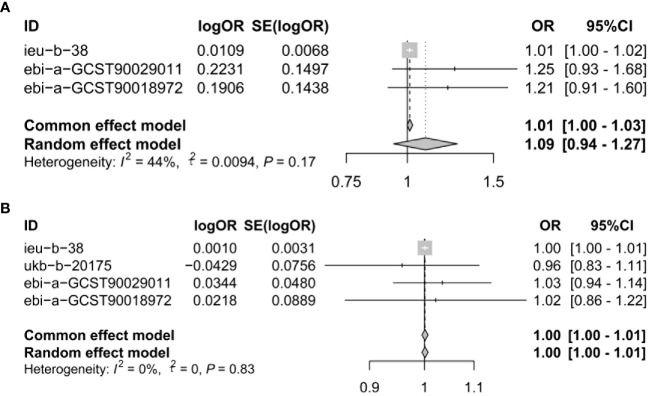
Meta-analysis was performed to assess the pooled effects of genetic proxied inhibition of **(A)** CACNA1D and **(B)** CACNB2 on depression. OR, odds ratio; CI, confidence interval.

To assure data reliability, we further meta-analyzed results across all database results of the IVW method, the detailed results are shown in **Figure 3**. Our meta-analysis results confirmed that there was no causal relationship between genetically proxied inhibition of CACNA1D and the risk of depression because the pooled confidence intervals crossed the null line (common effect model: OR = 1.01, 95% CI: 1.00-1.03; random effect model: OR = 1.09, 95% CI: 0.94-1.27). Additionally, the causal relationship between genetically proxied inhibition of CACNB2 and the risk of depression did not reach a significant level either (common effect model: OR = 1.00, 95% CI: 1.00-1.01; random effect model: OR = 1.00, 95% CI: 1.00-1.01).

## Discussion

In this study, we addressed whether genetically proxied CCBs play causal roles in depression by utilizing drug target MR approaches. Our findings indicated that no causal relationship between CCBs and the risk of depression onset in the European ancestry populations.

Exploring the causal relationship between exposure factors and diseases can provide crucial information for disease treatment and prevention. MR is a promising approach in human genetic research using genetic instruments as surrogates to assess the association between exposure and outcomes. In this drug-target MR analysis, a lack of association of genetic proxies for CCBs with depression was found. Findings from another recent study suggest that CACNB2, one of the drug targets of CCBs, was associated with a lower risk of depression in East Asian populations but not in the European populations (31). It was thus indicated that there may be a population-specific effect of CACNB2 polymorphisms on the risk of depression. Unlike that study, we selected genetic variants from multiple GWAS datasets as sources of instrumental variables for exposure to examined the effect for each gene on the outcome. Moreover, a large-scale GWAS summary statistics of CAD was used as a positive control outcome to ensure the effectiveness of instrumental variables. Finally, we pooled the IVW results from multiple database using meta-analysis. Our analysis using these approaches did not change the main conclusions. All these results and tests indicate that our findings are credible and robust.

Previous clinical studies undertaken to investigate the relationship between CCBs and depression risk were inconclusive. Based on the affinity and effects on the heart and artery, CCBs are classified as dihydropyridine CCBs (amlodipine, nifedipine, felodipine, nicardipine, etc.), which predominantly affect the vascular smooth muscle, and non-dihydropyridine CCBs (diltiazem, verapamil), which act mainly on myocardial L-type channels (32). There is evidence that different CCBs may have different effect on mood disorders. A recent nationwide study was conducted using the Danish population-based register to investigate whether commonly prescribed antihypertensive drugs were associated with depression (11). The investigators found that continued use of classes of CCBs, renin-angiotensin system agents, and β-blockers was associated with a decreased risk of depression, whereas diuretic use was not (11). Individual CCBs associated with decreased risk of depression included 3 of 10 CCBs: amlodipine, verapamil, and verapamil combinations, and no antihypertensive drugs were associated with an increased risk of depression (11). In another prospective cohort study, Tully et al. reported that participants who took a combination of selective serotonin reuptake inhibitors (SSRIs) with CCBs had improved depression scores compared with SSRIs combined with other antihypertensive agents (33). In contrast, Boal et al. used a large hospital database of 525,046 hypertensive patients from Scotland and revealed that patients taking CCBs had a higher risk of admission for mood disorders than patients taking renin-angiotensin system inhibitors at a 5-year follow-up (7). However, in the studies by Tully et al. and Boal et al., CCBs were discussed as a class of drugs, so specific impact of each drug may not be accurately assessed. In addition, a population-based cross-sectional study, enrolling 14,195 Australian and American older adults with hypertension but without other CVDs showed no associations between CCBs and depressive symptoms (34). The reasons for these inconsistent results may be attributable to differences in study designs, sample sizes, selection bias, or control for potential confounders. Our findings contradict some existing studies possibly because MR estimates for drug effects not fully corresponding to the results from RCTs with short-term follow-ups. The inherent heterogeneity of major depression might also be the cause of no causal associations in this study. Further RCTs with long-term follow-ups are required to validate the effects of CCBs on different subtypes of depression. An ongoing clinical trial of CCBs focusing on mood symptoms and cognition might provide additional evidence in the close future (35).

To date, much uncertainty exists about biological mechanisms underlying the association of CCBs and depression. It is worth noting that individual CCBs differ in their ability to cross the blood-brain barrier (36). Amlodipine is one of the most frequently prescribed antihypertensive agents and is not easily to cross the blood-brain barrier to some extent (36). This may partly explain the lack of association of CCBs with depression. However, the preclinical studies also produced some interesting findings. Animal studies on the effects of different classes of CCBs on depression have produced conflicting results. P-glycoprotein is a membrane transporter with a drug efflux function that contributes to the reduced bioavailability of some agents, including antidepressants. The experimental results confirmed that verapamil could play an antidepressant-like role by blocking the action of P-glycoprotein located in the blood–brain barrier (37). Conversely, in studies in which mice were acutely treated with CCBs, verapamil and diltiazem facilitated depression, perhaps by off-target inhibition of norepinephrine and serotonin release at higher dosages, whereas nifedipine exhibited an antidepressant-like behavior (38). Among different CCBs, individual drugs show some differences in pharmacological features, such as their relative preference for L-type voltage-gated calcium channel subtypes, their half-life and their permeability across the blood–brain barrier (39, 40). These factors might explain the different effects on mood state induced by individual CCBs. Furthermore, it is unclear whether these effects are mediated directly via central calcium channel antagonism or indirectly via cardiovascular effects or additional mechanisms. Therefore, further studies are necessary to clarify the exact mechanism.

Our study has several advantages. Firstly, by mimicking the genetic variation in targets of CCBs, the causal effects of drugs are inferred from the drug-target MR, avoiding confounding factors and reverse causality, as well as shortening experimental time and saving resources. Secondly, we used multiple GWAS datasets with relatively large sample size, which strengthens the validity of the study findings. Thirdly, genetic variations in genes encoding drug target proteins related to SBP were screened as surrogates for CCBs through standard screening procedures, and positive control analysis was performed in parallel to ensure the validity of the genetic instruments. Finally, sensitivity analysis was performed to assess the reliability and robustness of the results.

However, we acknowledge that this study has some limitations. Firstly, despite being termed a “natural randomized trial”, MR cannot completely substitute for randomized trials but rather provides supplementary information (41). Further high-quality RCTs should be conducted to confirm the association between CCBs and the risk of depression. Secondly, MR estimates represented long-term regulation of drug use on disease risk, and the results may have larger effect values than short-term effects of drug use in clinical trials. Thirdly, this study was restricted to individuals of European ancestry. The effects of CCBs may differ among different populations due to the genetic background among various ethnic groups. Hence, extrapolation of the results to other populations should be done with caution. Future studies are required to encompass a wider range of ancestries, such as African Americans, Hispanics, Caucasians, and other populations, to ensure the broader applicability of our results. Finally, we used genetic proxies for CCBs in general rather than distinct subtypes (dihydropyridine and non-dihydropyridine CCBs), although CCBs subtypes have a common mechanism of action by antagonizing L-type calcium channels in smooth muscle cells to reduce blood pressure. Thus, future studies are warranted to investigate the effect of CCBs subtypes on mood disorders.

## Conclusion

In conclusion, our MR study did not support a causal effect of CCBs on the risk of depression. To confirm the accuracy of our results, further MR studies based on large-scale GWAS data and clinical trials are needed to verify our findings. Given that CCBs are considered a first-line therapy for the treatment of hypertension, this finding adds support to the clinical safety of CCBs in daily practice.

## Data availability statement

The original contributions presented in the study are included in the article/**Supplementary Material**. Further inquiries can be directed to the corresponding author.

## Author contributions

CY: Conceptualization, Data curation, Investigation, Methodology, Resources, Supervision, Validation, Visualization, Writing – original draft, Writing – review & editing. TW: Conceptualization, Data curation, Formal analysis, Investigation, Methodology, Resources, Software, Validation, Visualization, Writing – review & editing. HW: Data curation, Methodology, Validation, Visualization, Writing – review & editing. GL: Formal analysis, Investigation, Validation, Writing – review & editing. LX: Conceptualization, Funding acquisition, Investigation, Project administration, Supervision, Validation, Writing – review & editing.

## Supplementary material

Supplementary Figure S1 Sensitivity analysis of genetic proxies for CCBs on CAD. **(A)** Leave-one-out analysis of CACNA1D inhibition (ieu-b-38) on CAD. **(B)** Leave-one-out analysis of CACNB2 inhibition (ieu-b-38) on CAD. **(C)** Leave-one-out analysis of CACNB3 inhibition (ieu-b-38) on CAD. **(D)** Leave-one-out analysis of CACNB2 inhibition (ukb-b-20175) on CAD. **(E)** Leave-one-out analysis of CACNA1D inhibition (ebi-a-GCST90029011) on CAD. **(F)** Leave-one-out analysis of CACNB2 inhibition (ebi-a-GCST90029011) on CAD. **(G)** Leave-one-out analysis of CACNA1D inhibition (ebi-a-GCST90018972) on CAD. **(H)** Leave-one-out analysis of CACNB2 inhibition (ebi-a-GCST90018972) on CAD.

Supplementary Figure S2 Sensitivity analysis of genetic proxies for CCBs on depression. **(A)** Leave-one-out analysis of CACNA1D inhibition (ieu-b-38) on depression. **(B)** Leave-one-out analysis of CACNB2 inhibition (ieu-b-38) on depression. **(C)** Leave-one-out analysis of CACNB3 inhibition (ieu-b-38) on depression. **(D)** Leave-one-out analysis of CACNB2 inhibition (ukb-b-20175) on depression. **(E)** Leave-one-out analysis of CACNA1D inhibition (ebi-a-GCST90029011) on depression. **(F)** Leave-one-out analysis of CACNB2 inhibition (ebi-a-GCST90029011) on depression. **(G)** Leave-one-out analysis of CACNA1D inhibition (ebi-a-GCST90018972) on depression. **(H)** Leave-one-out analysis of CACNB2 inhibition (ebi-a-GCST90018972) on depression.

## References

[1] GBD 2017 Risk Factor Collaborators. Global, regional, and national comparative risk assessment of 84 behavioural, environmental and occupational, and metabolic risks or clusters of risks for 195 countries and territories, 1990-2017: A systematic analysis for the global burden of disease study 2017. Lancet (London England). (2018) 392:1923–94. doi: 10.1016/s0140-6736(18)32225-6 PMC6227755 30496105

[2] Zhang B Zhang W Sun X Ge J Liu D. Physical comorbidity and health literacy mediate the relationship between social support and depression among patients with hypertension. Front Public Health. (2020) 8:304. doi: 10.3389/fpubh.2020.00304 32850572 PMC7419472

[3] Li Z Li Y Chen L Chen P Hu Y. Prevalence of depression in patients with hypertension: A systematic review and meta-analysis. Medicine. (2015) 94:e1317. doi: 10.1097/md.0000000000001317 26252317 PMC4616591

[4] Hiriscau EI Bodolea C. The role of depression and anxiety in frail patients with heart failure. Dis (Basel Switzerland). (2019) 7:45. doi: 10.3390/diseases7020045 PMC6631213 31248108

[5] Zhang Y Chen Y Ma L. Depression and cardiovascular disease in elderly: current understanding. J Clin Neurosci Off J Neurosurgical Soc Australasia. (2018) 47:1–5. doi: 10.1016/j.jocn.2017.09.022 29066229

[6] Elliott WJ Ram CV. Calcium channel blockers. J Clin hypertension (Greenwich Conn). (2011) 13:687–9. doi: 10.1111/jch.2011.13.issue-9 PMC8108866 21896151

[7] Boal AH Smith DJ McCallum L Muir S Touyz RM Dominiczak AF. Monotherapy with major antihypertensive drug classes and risk of hospital admissions for mood disorders. Hypertension (Dallas Tex 1979). (2016) 68:1132–8. doi: 10.1161/hypertensionaha.116.08188 PMC5058642 27733585

[8] Cao YY Xiang X Song J Tian YH Wang MY Wang XW. Distinct effects of antihypertensives on depression in the real-world setting: A retrospective cohort study. J Affect Disord. (2019) 259:386–91. doi: 10.1016/j.jad.2019.08.075 31470183

[9] Zhang L Bao Y Tao S Zhao Y Liu M. The association between cardiovascular drugs and depression/anxiety in patients with cardiovascular disease: A meta-analysis. Pharmacol Res. (2022) 175:106024. doi: 10.1016/j.phrs.2021.106024 34890773

[10] Li Y Fan Y Sun Y Alolga RN Xiao P Ma G. Antihypertensive drug use and the risk of depression: A systematic review and network meta-analysis. Front Pharmacol. (2021) 12:777987. doi: 10.3389/fphar.2021.777987 34819866 PMC8606787

[11] Kessing LV Rytgaard HC Ekstrøm CT Torp-Pedersen C Berk M Gerds TA. Antihypertensive drugs and risk of depression: A nationwide population-based study. Hypertension (Dallas Tex 1979). (2020) 76:1263–79. doi: 10.1161/hypertensionaha.120.15605 32829669

[12] Zabor EC Kaizer AM Hobbs BP. Randomized controlled trials. Chest. (2020) 158:S79–s87. doi: 10.1016/j.chest.2020.03.013 32658656 PMC8176647

[13] Zheng J Baird D Borges MC Bowden J Hemani G Haycock P. Recent developments in mendelian randomization studies. Curr Epidemiol Rep. (2017) 4:330–45. doi: 10.1007/s40471-017-0128-6 PMC5711966 29226067

[14] Larsson SC Bäck M Rees JMB Mason AM Burgess S. Body mass index and body composition in relation to 14 cardiovascular conditions in uk biobank: A mendelian randomization study. Eur Heart J. (2020) 41:221–6. doi: 10.1093/eurheartj/ehz388 PMC6945523 31195408

[15] Mokry LE Ahmad O Forgetta V Thanassoulis G Richards JB. Mendelian randomisation applied to drug development in cardiovascular disease: A review. J Med Genet. (2015) 52:71–9. doi: 10.1136/jmedgenet-2014-102438 25515070

[16] Bowden J Davey Smith G Burgess S. Mendelian randomization with invalid instruments: effect estimation and bias detection through egger regression. Int J Epidemiol. (2015) 44:512–25. doi: 10.1093/ije/dyv080 PMC4469799 26050253

[17] Skrivankova VW Richmond RC Woolf BAR Yarmolinsky J Davies NM Swanson SA. Strengthening the reporting of observational studies in epidemiology using mendelian randomization: the strobe-mr statement. Jama. (2021) 326:1614–21. doi: 10.1001/jama.2021.18236 34698778

[18] Evangelou E Warren HR Mosen-Ansorena D Mifsud B Pazoki R Gao H. Genetic analysis of over 1 million people identifies 535 new loci associated with blood pressure traits. Nat Genet. (2018) 50:1412–25. doi: 10.1038/s41588-018-0205-x PMC6284793 30224653

[19] Loh PR Kichaev G Gazal S Schoech AP Price AL. Mixed-model association for biobank-scale datasets. Nat Genet. (2018) 50:906–8. doi: 10.1038/s41588-018-0144-6 PMC6309610 29892013

[20] Sakaue S Kanai M Tanigawa Y Karjalainen J Kurki M Koshiba S. A cross-population atlas of genetic associations for 220 human phenotypes. Nat Genet. (2021) 53:1415–24. doi: 10.1038/s41588-021-00931-x PMC12208603 34594039

[21] Wishart DS Feunang YD Guo AC Lo EJ Marcu A Grant JR. Drugbank 5.0: A major update to the drugbank database for 2018. Nucleic Acids Res. (2018) 46:D1074–d82. doi: 10.1093/nar/gkx1037 PMC5753335 29126136

[22] Palmer TM Lawlor DA Harbord RM Sheehan NA Tobias JH Timpson NJ. Using multiple genetic variants as instrumental variables for modifiable risk factors. Stat Methods Med Res. (2012) 21:223–42. doi: 10.1177/0962280210394459 PMC3917707 21216802

[23] Burgess S Thompson SG. Bias in causal estimates from mendelian randomization studies with weak instruments. Stat Med. (2011) 30:1312–23. doi: 10.1002/sim.4197 21432888

[24] Howard DM Adams MJ Clarke TK Hafferty JD Gibson J Shirali M. Genome-wide meta-analysis of depression identifies 102 independent variants and highlights the importance of the prefrontal brain regions. Nat Neurosci. (2019) 22:343–52. doi: 10.1038/s41593-018-0326-7 PMC6522363 30718901

[25] van der Harst P Verweij N. Identification of 64 novel genetic loci provides an expanded view on the genetic architecture of coronary artery disease. Circ Res. (2018) 122:433–43. doi: 10.1161/circresaha.117.312086 PMC5805277 29212778

[26] Miyauchi K Kojima T Yokoyama T Kurata T Yokoyama K Kawamura M. Azelnidipine and amlodipine anti-coronary atherosclerosis trial in hypertensive patients undergoing coronary intervention by serial volumetric intravascular ultrasound analysis in juntendo university (Alps-J). Cardiovasc Drugs Ther. (2009) 23:409–13. doi: 10.1007/s10557-009-6192-5 19763803

[27] Yavorska OO Burgess S. Mendelianrandomization: an R package for performing mendelian randomization analyses using summarized data. Int J Epidemiol. (2017) 46:1734–9. doi: 10.1093/ije/dyx034 PMC5510723 28398548

[28] Burgess S Butterworth A Thompson SG. Mendelian randomization analysis with multiple genetic variants using summarized data. Genet Epidemiol. (2013) 37:658–65. doi: 10.1002/gepi.21758 PMC4377079 24114802

[29] Bowden J Del Greco MF Minelli C Davey Smith G Sheehan N Thompson J. A framework for the investigation of pleiotropy in two-sample summary data mendelian randomization. Stat Med. (2017) 36:1783–802. doi: 10.1002/sim.7221 PMC5434863 28114746

[30] Verbanck M Chen CY Neale B Do R. Detection of widespread horizontal pleiotropy in causal relationships inferred from mendelian randomization between complex traits and diseases. Nat Genet. (2018) 50:693–8. doi: 10.1038/s41588-018-0099-7 PMC6083837 29686387

[31] Fan B Zhao JV. Genetic proxies for antihypertensive drugs and mental disorders: mendelian randomization study in european and east asian populations. BMC Med. (2024) 22:6. doi: 10.1186/s12916-023-03218-6 38166843 PMC10763027

[32] Zhao T Jiang W Zhen X Jin C Zhang Y Li H. Quechers-based approach to the extraction of five calcium channel blockers from plasma determined by uplc-ms/ms. Molecules (Basel Switzerland). (2023) 28:671. doi: 10.3390/molecules28020671 36677729 PMC9866929

[33] Tully PJ Peters R Pérès K Anstey KJ Tzourio C. Effect of ssri and calcium channel blockers on depression symptoms and cognitive function in elderly persons treated for hypertension: three city cohort study. Int psychogeriatrics. (2018) 30:1345–54. doi: 10.1017/s1041610217002903 29559030

[34] Agustini B Mohebbi M Woods RL McNeil JJ Nelson MR Shah RC. The association of antihypertensive use and depressive symptoms in a large older population with hypertension living in Australia and the United States: A cross-sectional study. J Hum hypertension. (2020) 34:787–94. doi: 10.1038/s41371-020-0303-y PMC7390661 32001828

[35] Atkinson LZ Colbourne L Smith A Harmer CH Nobre AC Rendell J. The oxford study of calcium channel antagonism, cognition, mood instability and sleep (Oxcams): study protocol for a randomised controlled, experimental medicine study. Trials. (2019) 20:120. doi: 10.1186/s13063-019-3175-0 30755265 PMC6373140

[36] Colbourne L Harrison PJ. Brain-penetrant calcium channel blockers are associated with a reduced incidence of neuropsychiatric disorders. Mol Psychiatry. (2022) 27:3904–12. doi: 10.1038/s41380-022-01615-6 PMC9708561 35618884

[37] Clarke G O'Mahony SM Cryan JF Dinan TG. Verapamil in treatment resistant depression: A role for the P-glycoprotein transporter? Hum Psychopharmacol. (2009) 24:217–23. doi: 10.1002/hup.1008 19212940

[38] Srivastava SK Nath C. The differential effects of calcium channel blockers in the behavioural despair test in mice. Pharmacol Res. (2000) 42:293–7. doi: 10.1006/phrs.2000.0696 10987986

[39] Cipriani A Saunders K Attenburrow MJ Stefaniak J Panchal P Stockton S. A systematic review of calcium channel antagonists in bipolar disorder and some considerations for their future development. Mol Psychiatry. (2016) 21:1324–32. doi: 10.1038/mp.2016.86 PMC5030455 27240535

[40] Zamponi GW Striessnig J Koschak A Dolphin AC. The physiology, pathology, and pharmacology of voltage-gated calcium channels and their future therapeutic potential. Pharmacol Rev. (2015) 67:821–70. doi: 10.1124/pr.114.009654 PMC4630564 26362469

[41] Carugo S Sirtori CR Corsini A Tokgozoglu L Ruscica M. Pcsk9 inhibition and risk of diabetes: should we worry? Curr Atheroscl Rep. (2022) 24:995–1004. doi: 10.1007/s11883-022-01074-y PMC9750910 36383291

