# Supplementary figures and images for: Causal relationship between genetic proxies for calcium channel blockers and the risk of depression: a drug-target Mendelian randomization study
Source: Front Psychiatry. 2024 May 10;15:1377705. doi: 10.3389/fpsyt.2024.1377705 (PMC11117141; doi:10.3389/fpsyt.2024.1377705)

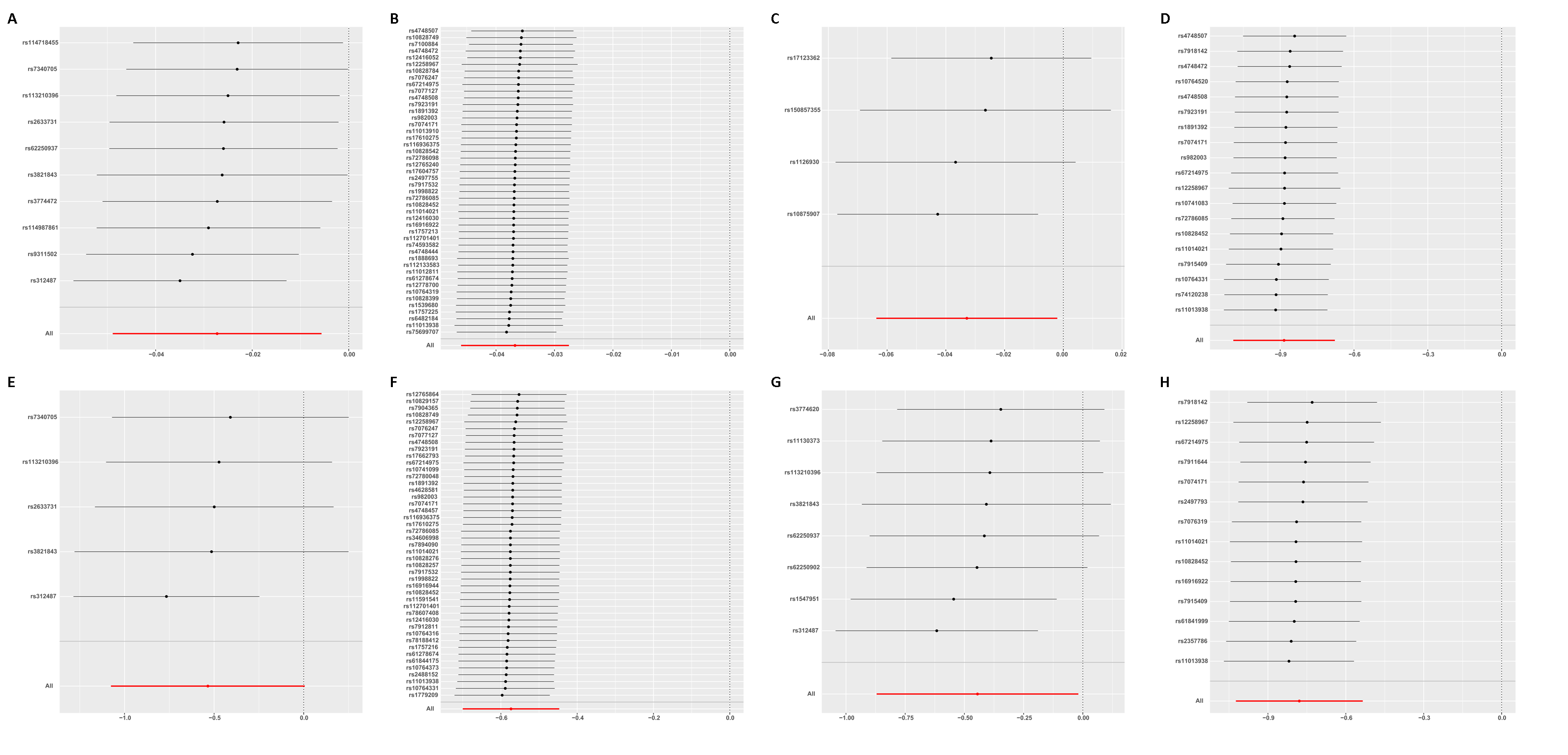

Supplement: Supplementary Figure S1 — Sensitivity analysis of genetic proxies for CCBs on CAD. (A) Leave-one-out analysis of CACNA1D inhibition (ieu-b-38) on CAD. (B) Leave-one-out analysis of CACNB2 inhibition (ieu-b-38) on CAD. (C) Leave-one-out analysis of CACNB3 inhibition (ieu-b-38) on CAD. (D) Leave-one-out analysis of CACNB2 inhibition (ukb-b-20175) on CAD. (E) Leave-one-out analysis of CACNA1D inhibition (ebi-a-GCST90029011) on CAD. (F) Leave-one-out analysis of CACNB2 inhibition (ebi-a-GCST90029011) on CAD. (G) Leave-one-out analysis of CACNA1D inhibition (ebi-a-GCST90018972) on CAD. (H) Leave-one-out analysis of CACNB2 inhibition (ebi-a-GCST90018972) on CAD. [file Image_1.tif]

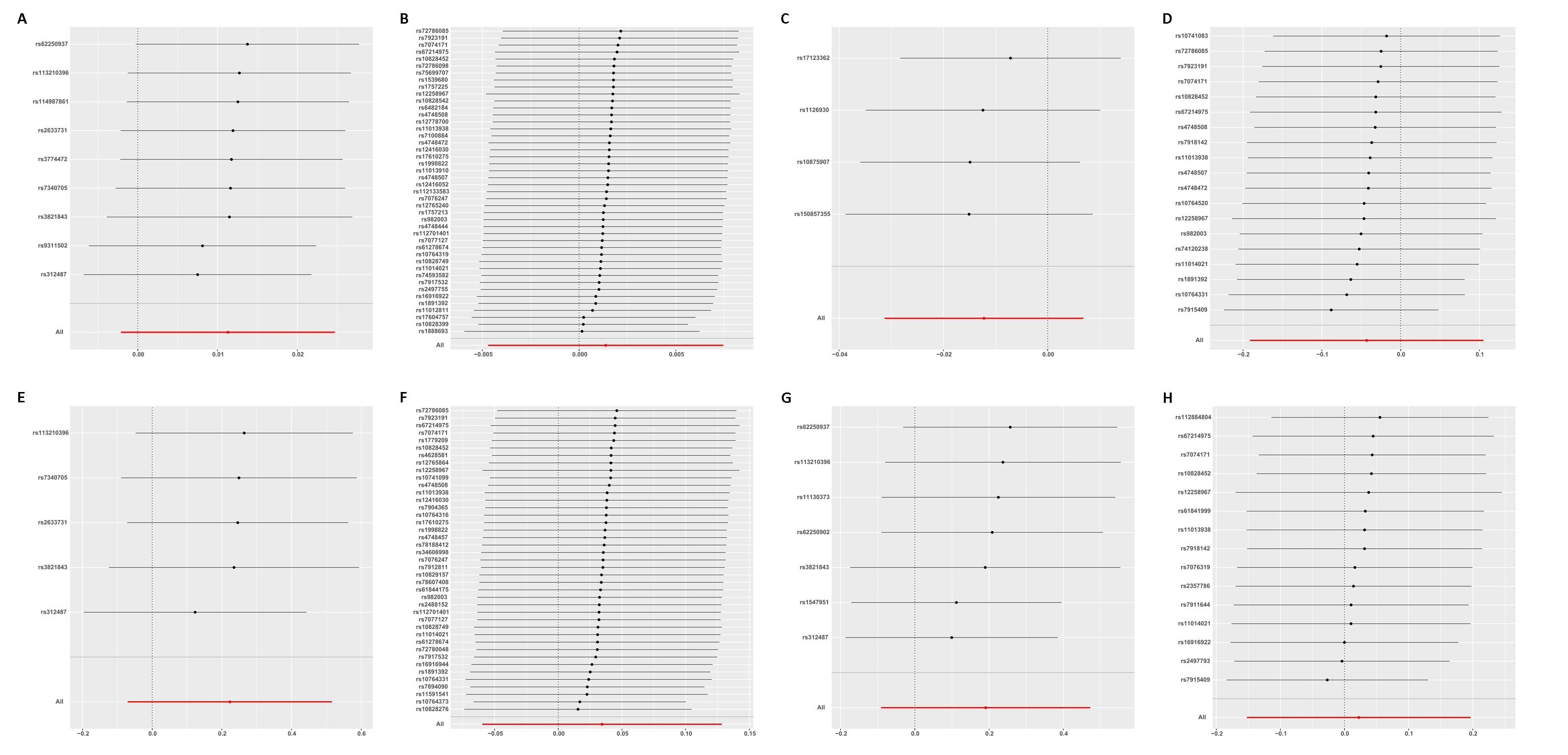

Supplement: Supplementary Figure S2 — Sensitivity analysis of genetic proxies for CCBs on depression. (A) Leave-one-out analysis of CACNA1D inhibition (ieu-b-38) on depression. (B) Leave-one-out analysis of CACNB2 inhibition (ieu-b-38) on depression. (C) Leave-one-out analysis of CACNB3 inhibition (ieu-b-38) on depression. (D) Leave-one-out analysis of CACNB2 inhibition (ukb-b-20175) on depression. (E) Leave-one-out analysis of CACNA1D inhibition (ebi-a-GCST90029011) on depression. (F) Leave-one-out analysis of CACNB2 inhibition (ebi-a-GCST90029011) on depression. (G) Leave-one-out analysis of CACNA1D inhibition (ebi-a-GCST90018972) on depression. (H) Leave-one-out analysis of CACNB2 inhibition (ebi-a-GCST90018972) on depression. [file Image_2.tif]
