# Supplementary material for: Causal relationship between genetic proxies for calcium channel blockers and the risk of depression: a drug-target Mendelian randomization study
Source: Front Psychiatry. 2024 May 10;15:1377705. doi: 10.3389/fpsyt.2024.1377705 (PMC11117141; doi:10.3389/fpsyt.2024.1377705)
Supplement: Supplementary file 3 [file Table_1.docx]

| Table S1. STROBE-MR Checklist | | | | |
| --- | --- | --- | --- | --- |
| Item No. | Section | Checklist item | Page No. | Relevant text from manuscript |
| Title and abstract | | | | |
| 1 | Title and abstract | Indicate mendelian randomization (MR) as the study’s design in the title and/or the abstract if that is a main purpose of the study. | 1-2 | Title and Abstract |
| Introduction | | | | |
| 2 | Background | Explain the scientific background and rationale for the reported study. What is the exposure? Is a potential causal relationship between exposure and outcome plausible? Justify why MR is a helpful method to address the study question. | 2 | Introduction: paragraphs 1-3 |
| 3 | Objectives | State specific objectives clearly, including prespecified causal hypotheses (if any). State that MR is a method that, under specific assumptions, intends to estimate causal effects. | 2 | Introduction: paragraph 3 |
| Methods | | | | |
| 4 | Study design and data sources | Present key elements of the study design early in the article. Consider including a table listing sources of data for all phases of the study. For each data source contributing to the analysis, describe the following: |  |  |
|  | a | Setting: Describe the study design and the underlying population, if possible. Describe the setting, locations, and relevant dates, including periods of recruitment, exposure, follow-up, and data collection, when available. | 2-4 | Materials and methods: paragraphs 2-4 |
|  | b | Participants: Report the eligibility criteria and the sources and methods of selection of participants. Report the sample size and whether any power or sample size calculations were carried out prior to the main analysis. | 3-4 | Materials and methods: paragraphs 3-4 |
|  | c | Describe measurement, quality control, and selection of genetic variants. | 3-4 | Materials and methods: paragraph 3 |
|  | d | For each exposure, outcome, and other relevant variables, describe methods of assessment and diagnostic criteria for diseases. | 3-4 | Materials and methods: paragraphs 3-4 |
|  | e | Provide details of ethics committee approval and participant informed consent, if relevant. | 2 | Materials and methods: paragraph 1 |
| 5 | Assumptions | Explicitly state the 3 core instrumental variable (IV) assumptions for the main analysis (relevance, independence, and exclusion restriction), as well assumptions for any additional or sensitivity analysis. | 2 | Materials and methods: paragraph 2 |
| 6 | Statistical methods: main analysis | Describe statistical methods and statistics used. |  |  |
|  | a | Describe how quantitative variables were handled in the analyses (i.e., scale, units, model). | 3-4 | Materials and methods: paragraphs 3-5 |
|  | b | Describe how genetic variants were handled in the analyses and, if applicable, how their weights were selected. | 3-4 | Materials and methods: paragraph 3 |
|  | c | Describe the MR estimator (e.g., 2-stage least squares, Wald ratio) and related statistics. Detail the included covariates and, in case of 2-sample MR, whether the same covariate set was used for adjustment in the 2 samples. | 4 | Materials and methods: paragraph 5 |
|  | d | Explain how missing data were addressed. |  | N/A |
|  | e | If applicable, indicate how multiple testing was addressed. |  | N/A |
| 7 | Assessment of assumptions | Describe any methods or prior knowledge used to assess the assumptions or justify their validity. | 4 | Materials and methods: paragraph 5 |
| 8 | Sensitivity analyses and additional analyses | Describe any sensitivity analyses or additional analyses performed (e.g., comparison of effect estimates from different approaches, independent replication, bias analytic techniques, validation of instruments, simulations). | 4 | Materials and methods: paragraph 5 |
| 9 | Software and preregistration |  |  |  |
|  | a | Name statistical software and package(s), including version and settings used. | 4 | Materials and methods: paragraph 5 |
|  | b | State whether the study protocol and details were preregistered (as well as when and where). |  | N/A |
| Results | | | | |
| 10 | Descriptive data |  |  |  |
|  | a | Report the numbers of individuals at each stage of included studies and reasons for exclusion. Consider use of a flow diagram. | 3-4 | Materials and methods: paragraphs 3-4 |
|  | b | Report summary statistics for phenotypic exposure(s), outcome(s), and other relevant variables (e.g., means, SDs, proportions). |  | Table S3, Table S4, Table S5 and Table S6 |
|  | c | If the data sources include meta-analyses of previous studies, provide the assessments of heterogeneity across these studies. |  | N/A |
|  | d | For 2-sample MR: i. Provide justification of the similarity of the genetic variant–exposure associations between the exposure and outcome samples. ii. Provide information on the number of individuals who overlap between the exposure and outcome studies. | 3-4 | Materials and methods: paragraphs 3-4 |
| 11 | Main results |  |  |  |
|  | a | Report the associations between genetic variant and exposure and between genetic variant and outcome, preferably on an interpretable scale. |  | Table S3, Table S4, Table S5 and Table S6 |
|  | b | Report MR estimates of the relationship between exposure and outcome and the measures of uncertainty from the MR analysis, on an interpretable scale, such as odds ratio or relative risk per SD difference. | 4-5 | Results: paragraphs 1-2 and Figure 2 |
|  | c | If relevant, consider translating estimates of relative risk into absolute risk for a meaningful time period. |  | N/A |
|  | d | Consider plots to visualize results (e.g., forest plot, scatterplot of associations between genetic variants and outcome vs between genetic variants and exposure). | 5 | Figure 2 |
| 12 | Assessment of assumptions |  |  |  |
|  | a | Report the assessment of the validity of the assumptions. | 4-5 | Results: paragraphs 1-2 and Figure 2 |
|  | b | Report any additional statistics (e.g., assessments of heterogeneity across genetic variants, such as I2, Q statistic, or E-value). | 5-6 | Results: paragraph 3 and Table 2 |
| 13 | Sensitivity analyses and additional analyses |  |  |  |
|  | a | Report any sensitivity analyses to assess the robustness of the main results to violations of the assumptions. | 4-5 | Results: paragraphs 1-2 and Figure 2 |
|  | b | Report results from other sensitivity analyses or additional analyses. |  | Figure S1 and Figure S2 |
|  | c | Report any assessment of the direction of the causal relationship (e.g., bidirectional MR). |  | N/A |
|  | d | When relevant, report and compare with estimates from non-MR analyses. |  | N/A |
|  | e | Consider additional plots to visualize results (e.g., leave-one-out analyses). |  | Figure S1 and Figure S2 |
| Discussion | | | | |
| 14 | Key results | Summarize key results with reference to study objectives. | 5 | Discussion: paragraph 1 |
| 15 | Limitations | Discuss limitations of the study, taking into account the validity of the IV assumptions, other sources of potential bias, and imprecision. Discuss both direction and magnitude of any potential bias and any efforts to address them. | 7 | Discussion: paragraph 6 |
| 16 | Interpretation |  |  |  |
|  | a | Meaning: Give a cautious overall interpretation of results in the context of their limitations and in comparison with other studies. | 5-7 | Discussion: paragraphs 2-4 |
|  | b | Mechanism: Discuss underlying biological mechanisms that could drive a potential causal relationship between the investigated exposure and the outcome, and whether the gene-environment equivalence assumption is reasonable. Use causal language carefully, clarifying that IV estimates may provide causal effects only under certain assumptions. | 5-7 | Discussion: paragraphs 2-4 |
|  | c | Clinical relevance: Discuss whether the results have clinical or public policy relevance, and to what extent they inform effect sizes of possible interventions. | 5-7 | Discussion: paragraphs 2-4 |
| 17 | Generalizability | Discuss the generalizability of the study results (a) to other populations, (b) across other exposure periods/timings, and (c) across other levels of exposure. | 7 | Discussion: paragraph 6 |
| Other Information | | | | |
| 18 | Funding | Describe sources of funding and the role of funders in the present study and, if applicable, sources of funding for the databases and original study or studies on which the present study is based. | 8 | Funding information |
| 19 | Data and data sharing | Provide the data used to perform all analyses or report where and how the data can be accessed, and reference these sources in the article. Provide the statistical code needed to reproduce the results in the article or report whether the code is publicly accessible and, if so, where. | 7 | Data availability statement |
| 20 | Conflicts of interest | All authors should declare all potential conflicts of interest. | 8 | Conflict of interest |

Table S2. Gene regions for the drug targets and the corresponding SNP number

| Gene | Gene position (GRCh37/hg19) | SNP number | | | | |
| --- | --- | --- | --- | --- | --- | --- |
|  |  | ieu-b-38 | ukb-b-20175 | ebi-a-GCST90029011 | ebi-a-GCST90018972 |  |
| CACNA1C | Chr12: 2162153–2807116 | 2 | 0 | 0 | 0 |  |
| CACNA1D | Chr3: 53528638–53847760 | 10 | 1 | 5 | 8 |  |
| CACNA1F | ChrX: 49061523–49089802 | 0 | 0 | 0 | 0 |  |
| CACNA1S | Chr1: 201008640–201081554 | 0 | 0 | 0 | 0 |  |
| CACNB1 | Chr17: 37329706–37353922 | 0 | 0 | 0 | 0 |  |
| CACNB2 | Chr10: 18429353–18832486 | 46 | 19 | 45 | 15 |  |
| CACNB3 | Chr12: 49208263–49222724 | 4 | 1 | 2 | 1 |  |
| CACNB4 | Chr2: 152689285–152955681 | 0 | 0 | 0 | 0 |  |
| CACNG1 | Chr17: 65040670–65052913 | 0 | 0 | 0 | 0 |  |
| CACNA2D1 | Chr7: 81575760–82073272 | 0 | 0 | 0 | 0 |  |
| CACNA2D2 | Chr3: 50400044–50541675 | 0 | 0 | 0 | 0 |  |

Note: Gene position was obtained by searching the database Gene of NCBI. Abbreviations: Chr, chromosome.

Table S3. Details of SNPs corresponding to drug target genes (ieu-b-38)

| Gene | SNPs | Chr | Position | Beta | SE | *P* value | Effect alleles | Other alleles | R^2^ | F |
| --- | --- | --- | --- | --- | --- | --- | --- | --- | --- | --- |
| CACNA1C | rs2239046 | 12 | 2434419 | 0.2082 | 0.0322 | 9.58E-11 | A | G | 5.66E-05 | 41.80695 |
|  | rs714277 | 12 | 2514270 | 0.1986 | 0.0333 | 2.38E-09 | T | C | 4.81E-05 | 35.56877 |
| CACNA1D | rs113210396 | 3 | 53612327 | -0.4338 | 0.077 | 1.76E-08 | T | G | 4.78E-05 | 31.73923 |
|  | rs114718455 | 3 | 53464055 | 0.5102 | 0.0905 | 1.72E-08 | G | A | 4.96E-05 | 31.78209 |
|  | rs114987861 | 3 | 53605712 | 0.5289 | 0.0958 | 3.36E-08 | A | G | 4.48E-05 | 30.47999 |
|  | rs2633731 | 3 | 53738424 | 0.1963 | 0.0309 | 2.21E-10 | C | T | 5.56E-05 | 40.35733 |
|  | rs312487 | 3 | 53545622 | -0.2194 | 0.0307 | 9.65E-13 | C | T | 7.23E-05 | 51.07346 |
|  | rs3774472 | 3 | 53638200 | 0.1747 | 0.0303 | 8.21E-09 | G | A | 4.59E-05 | 33.24293 |
|  | rs3821843 | 3 | 53558012 | 0.3373 | 0.0335 | 6.56E-24 | A | G | 1.48E-04 | 101.3776 |
|  | rs62250937 | 3 | 53870318 | -0.3238 | 0.056 | 7.28E-09 | C | T | 5.10E-05 | 33.43307 |
|  | rs7340705 | 3 | 53734443 | 0.2425 | 0.0322 | 4.87E-14 | C | T | 7.79E-05 | 56.71665 |
|  | rs9311502 | 3 | 53560321 | 0.2463 | 0.0355 | 3.87E-12 | C | T | 6.62E-05 | 48.1361 |
| CACNB2 | rs10764319 | 10 | 18428415 | 0.2693 | 0.0329 | 2.54E-16 | T | C | 9.19E-05 | 67.00076 |
|  | rs10828399 | 10 | 18553968 | -0.1947 | 0.0302 | 1.10E-10 | A | G | 5.67E-05 | 41.56395 |
|  | rs10828452 | 10 | 18592450 | -0.3046 | 0.0388 | 4.20E-15 | T | A | 9.07E-05 | 61.63041 |
|  | rs10828542 | 10 | 18627285 | -0.1817 | 0.0311 | 5.18E-09 | G | A | 4.71E-05 | 34.13405 |
|  | rs10828749 | 10 | 18756881 | -0.3658 | 0.0309 | 2.27E-32 | A | G | 1.95E-04 | 140.1423 |
|  | rs10828784 | 10 | 18788273 | -0.2021 | 0.0345 | 4.49E-09 | G | C | 5.42E-05 | 34.31572 |
|  | rs11012811 | 10 | 18438456 | 0.3095 | 0.0326 | 2.31E-21 | T | G | 1.23E-04 | 90.13323 |
|  | rs11013910 | 10 | 18663370 | 0.2672 | 0.0449 | 2.70E-09 | A | G | 4.86E-05 | 35.41433 |
|  | rs11013938 | 10 | 18669271 | -0.3265 | 0.035 | 1.17E-20 | C | G | 1.22E-04 | 87.022 |
|  | rs11014021 | 10 | 18682233 | -0.3245 | 0.0307 | 4.29E-26 | A | T | 1.54E-04 | 111.7253 |
|  | rs112133583 | 10 | 18695681 | -0.5546 | 0.0973 | 1.18E-08 | T | C | 5.20E-05 | 32.48872 |
|  | rs112701401 | 10 | 18644811 | -0.5026 | 0.0921 | 4.91E-08 | G | C | 4.46E-05 | 29.77998 |
|  | rs116936375 | 10 | 18737135 | -0.5739 | 0.081 | 1.40E-12 | A | G | 7.45E-05 | 50.1997 |
|  | rs12258967 | 10 | 18727959 | -0.6327 | 0.0337 | 1.08E-78 | G | C | 4.95E-04 | 352.4801 |
|  | rs12416030 | 10 | 18789075 | 0.2088 | 0.0381 | 4.32E-08 | C | T | 4.23E-05 | 30.03377 |
|  | rs12416052 | 10 | 18789267 | -0.1987 | 0.0311 | 1.59E-10 | C | T | 5.69E-05 | 40.82007 |
|  | rs12765240 | 10 | 18438372 | 0.396 | 0.052 | 2.52E-14 | T | C | 7.93E-05 | 57.99392 |
|  | rs12778700 | 10 | 18385490 | -0.1972 | 0.033 | 2.24E-09 | C | T | 5.14E-05 | 35.70958 |
|  | rs1539680 | 10 | 18502889 | -0.3259 | 0.0375 | 3.37E-18 | C | G | 1.04E-04 | 75.52748 |
|  | rs16916922 | 10 | 18467744 | 0.3662 | 0.0433 | 2.86E-17 | T | A | 9.78E-05 | 71.5253 |
|  | rs1757213 | 10 | 18537594 | -0.3084 | 0.0507 | 1.15E-09 | G | A | 5.53E-05 | 37.00083 |
|  | rs1757225 | 10 | 18516925 | -0.2453 | 0.0325 | 4.29E-14 | G | A | 7.80E-05 | 56.9675 |
|  | rs17604757 | 10 | 18442940 | 0.5022 | 0.0606 | 1.12E-16 | G | A | 9.47E-05 | 68.67631 |
|  | rs17610275 | 10 | 18621630 | -0.3868 | 0.0613 | 2.87E-10 | G | T | 5.95E-05 | 39.81536 |
|  | rs1888693 | 10 | 18440444 | 0.3858 | 0.0317 | 4.69E-34 | A | G | 2.02E-04 | 148.1169 |
|  | rs1891392 | 10 | 18336421 | -0.2276 | 0.0334 | 9.02E-12 | C | T | 6.35E-05 | 46.43553 |
|  | rs1998822 | 10 | 18755664 | 0.1958 | 0.0343 | 1.15E-08 | G | A | 4.61E-05 | 32.58636 |
|  | rs2497755 | 10 | 18282667 | -0.303 | 0.0493 | 7.72E-10 | T | A | 5.16E-05 | 37.77376 |
|  | rs4748444 | 10 | 18494482 | -0.1939 | 0.0327 | 3.13E-09 | C | T | 5.02E-05 | 35.16081 |
|  | rs4748472 | 10 | 18776197 | 0.3161 | 0.0319 | 4.04E-23 | T | C | 1.35E-04 | 98.18981 |
|  | rs4748507 | 10 | 18957845 | 0.2216 | 0.0367 | 1.55E-09 | T | A | 4.99E-05 | 36.45912 |
|  | rs4748508 | 10 | 18958646 | 0.1887 | 0.0302 | 4.37E-10 | G | A | 5.35E-05 | 39.0417 |
|  | rs61278674 | 10 | 18481737 | 0.3298 | 0.054 | 1.03E-09 | G | A | 5.52E-05 | 37.30031 |
|  | rs6482184 | 10 | 18403118 | 0.1949 | 0.0316 | 6.65E-10 | C | T | 5.34E-05 | 38.04068 |
|  | rs67214975 | 10 | 18727251 | -0.4144 | 0.0307 | 1.42E-41 | A | C | 2.55E-04 | 182.2055 |
|  | rs7074171 | 10 | 18676897 | 0.3061 | 0.0314 | 1.76E-22 | G | A | 1.37E-04 | 95.03118 |
|  | rs7076247 | 10 | 18759629 | -0.2557 | 0.0309 | 1.33E-16 | C | T | 9.29E-05 | 68.47678 |
|  | rs7077127 | 10 | 18717348 | 0.217 | 0.0334 | 7.95E-11 | T | G | 5.77E-05 | 42.21097 |
|  | rs7100884 | 10 | 18812697 | 0.2064 | 0.0367 | 1.85E-08 | G | A | 4.32E-05 | 31.62904 |
|  | rs72786085 | 10 | 18713206 | -0.5309 | 0.0595 | 4.46E-19 | C | G | 1.20E-04 | 79.61414 |
|  | rs72786098 | 10 | 18729855 | -0.5033 | 0.0883 | 1.18E-08 | A | G | 4.61E-05 | 32.48862 |
|  | rs74593582 | 10 | 18374059 | 0.7581 | 0.1349 | 1.91E-08 | C | T | 5.12E-05 | 31.58113 |
|  | rs75699707 | 10 | 18359294 | -0.5761 | 0.1028 | 2.10E-08 | A | G | 4.90E-05 | 31.40568 |
|  | rs7917532 | 10 | 18373902 | -0.2323 | 0.0304 | 2.01E-14 | C | T | 8.07E-05 | 58.39156 |
|  | rs7923191 | 10 | 18727901 | 0.369 | 0.0376 | 1.09E-22 | G | A | 1.35E-04 | 96.31099 |
|  | rs982003 | 10 | 18707296 | -0.2414 | 0.0351 | 6.21E-12 | T | C | 6.44E-05 | 47.29978 |
| CACNB3 | rs10875907 | 12 | 49389410 | -0.7158 | 0.1219 | 4.28E-09 | A | G | 5.55E-05 | 34.48058 |
|  | rs1126930 | 12 | 49399132 | 0.6378 | 0.0856 | 9.48E-14 | C | G | 8.11E-05 | 55.51626 |
|  | rs150857355 | 12 | 49209340 | 0.9406 | 0.1122 | 5.20E-17 | C | G | 1.08E-04 | 70.27857 |
|  | rs17123362 | 12 | 49255964 | 0.4067 | 0.0672 | 1.41E-09 | A | G | 5.34E-05 | 36.62761 |

Abbreviations: Chr, chromosome; SE, standard error; SNPs, single nucleotide polymorphisms; R^2^, proportion of variation explained; F, F statistics.

Table S4. Details of SNPs corresponding to drug target genes (ukb-b-20175)

| Gene | SNPs | chr | Position | Beta | SE | *P* value | Effect alleles | Other alleles | R^2^ | F |
| --- | --- | --- | --- | --- | --- | --- | --- | --- | --- | --- |
| CACNA1D | rs35593046 | 3 | 53553923 | -0.01325 | 0.002372 | 2.30E-08 | T | G | 7.15E-05 | 31.19533 |
| CACNB2 | rs10741083 | 10 | 18790858 | 0.013735 | 0.002154 | 1.80E-10 | C | T | 9.32E-05 | 40.66367 |
|  | rs10764331 | 10 | 18451836 | 0.016738 | 0.002111 | 2.20E-15 | G | A | 1.44E-04 | 62.89141 |
|  | rs10764520 | 10 | 18758481 | -0.01198 | 0.002136 | 2.00E-08 | C | T | 7.21E-05 | 31.45736 |
|  | rs10828452 | 10 | 18592450 | -0.01495 | 0.002613 | 1.10E-08 | T | A | 7.50E-05 | 32.73495 |
|  | rs11013938 | 10 | 18669271 | -0.01479 | 0.002383 | 5.40E-10 | C | G | 8.83E-05 | 38.54391 |
|  | rs11014021 | 10 | 18682233 | -0.01547 | 0.002124 | 3.20E-13 | A | T | 1.22E-04 | 53.08808 |
|  | rs12258967 | 10 | 18727959 | -0.03018 | 0.002268 | 2.10E-40 | G | C | 4.06E-04 | 177.071 |
|  | rs1891392 | 10 | 18336421 | -0.01256 | 0.002277 | 3.50E-08 | C | T | 6.97E-05 | 30.42029 |
|  | rs4748472 | 10 | 18776197 | 0.015282 | 0.002195 | 3.40E-12 | T | C | 1.11E-04 | 48.46178 |
|  | rs4748507 | 10 | 18957845 | 0.014354 | 0.002504 | 9.90E-09 | T | A | 7.53E-05 | 32.86668 |
|  | rs4748508 | 10 | 18958646 | 0.013082 | 0.00208 | 3.20E-10 | G | A | 9.06E-05 | 39.55516 |
|  | rs67214975 | 10 | 18727251 | -0.02107 | 0.002086 | 5.40E-24 | A | C | 2.34E-04 | 102.0671 |
|  | rs7074171 | 10 | 18676897 | 0.013397 | 0.002139 | 3.80E-10 | G | A | 8.98E-05 | 39.20706 |
|  | rs72786085 | 10 | 18713206 | -0.02284 | 0.003799 | 1.80E-09 | C | G | 8.28E-05 | 36.15461 |
|  | rs74120238 | 10 | 18451689 | 0.017418 | 0.002885 | 1.60E-09 | A | G | 8.35E-05 | 36.43943 |
|  | rs7915409 | 10 | 18419595 | 0.033683 | 0.004344 | 8.90E-15 | A | G | 1.38E-04 | 60.12029 |
|  | rs7918142 | 10 | 18755840 | -0.0187 | 0.002102 | 5.70E-19 | G | T | 1.81E-04 | 79.16786 |
|  | rs7923191 | 10 | 18727901 | 0.017231 | 0.002596 | 3.20E-11 | G | A | 1.01E-04 | 44.05085 |
|  | rs982003 | 10 | 18707296 | -0.01422 | 0.002421 | 4.30E-09 | T | C | 7.90E-05 | 34.48535 |
| CACNB3 | rs138650910 | 12 | 49305787 | 0.037092 | 0.005656 | 5.40E-11 | T | A | 9.86E-05 | 43.01389 |

Abbreviations: Chr, chromosome; SE, standard error; SNPs, single nucleotide polymorphisms; R^2^, proportion of variation explained; F, F statistics.

Table S5. Details of SNPs corresponding to drug target genes (ebi-a-GCST90029011)

| Gene | SNPs | chr | Position | Beta | SE | *P* value | Effect alleles | Other alleles | R^2^ | F |
| --- | --- | --- | --- | --- | --- | --- | --- | --- | --- | --- |
| CACNA1D | rs113210396 | 3 | 53612327 | -0.02377 | 0.004895 | 4.30E-08 | T | G | 5.02E-05 | 23.57566 |
|  | rs2633731 | 3 | 53738424 | 0.011207 | 0.002099 | 2.30E-08 | C | T | 6.07E-05 | 28.50286 |
|  | rs312487 | 3 | 53545622 | -0.0117 | 0.00207 | 2.60E-09 | C | T | 6.80E-05 | 31.95202 |
|  | rs3821843 | 3 | 53558012 | 0.018118 | 0.002222 | 4.80E-17 | A | G | 1.42E-04 | 66.50368 |
|  | rs7340705 | 3 | 53734443 | 0.015329 | 0.002198 | 5.00E-13 | C | T | 1.04E-04 | 48.65252 |
| CACNB2 | rs10741099 | 10 | 18820341 | 0.014846 | 0.002052 | 1.20E-13 | T | C | 1.11E-04 | 52.32587 |
|  | rs10764316 | 10 | 18409507 | 0.018082 | 0.002361 | 3.70E-15 | G | C | 1.25E-04 | 58.64377 |
|  | rs10764331 | 10 | 18451836 | 0.024797 | 0.002079 | 7.40E-34 | G | A | 3.03E-04 | 142.2964 |
|  | rs10764373 | 10 | 18553665 | -0.01265 | 0.002048 | 6.00E-10 | T | G | 8.12E-05 | 38.16507 |
|  | rs10828257 | 10 | 18410612 | 0.019109 | 0.003375 | 2.00E-08 | T | G | 6.82E-05 | 32.05346 |
|  | rs10828276 | 10 | 18443616 | 0.035164 | 0.004156 | 2.80E-17 | A | C | 1.52E-04 | 71.60153 |
|  | rs10828452 | 10 | 18592450 | -0.02 | 0.002572 | 5.10E-15 | T | A | 1.29E-04 | 60.42371 |
|  | rs10828749 | 10 | 18756881 | -0.02374 | 0.002098 | 6.00E-31 | A | G | 2.73E-04 | 128.1002 |
|  | rs10829157 | 10 | 18951463 | 0.015539 | 0.002513 | 4.00E-10 | A | G | 8.14E-05 | 38.23796 |
|  | rs11013938 | 10 | 18669271 | -0.01999 | 0.002347 | 6.50E-18 | C | G | 1.54E-04 | 72.51986 |
|  | rs11014021 | 10 | 18682233 | -0.01954 | 0.002092 | 1.70E-21 | A | T | 1.86E-04 | 87.28156 |
|  | rs112701401 | 10 | 18644811 | -0.03529 | 0.006023 | 3.40E-09 | G | C | 7.31E-05 | 34.34375 |
|  | rs11591541 | 10 | 18421314 | 0.027493 | 0.002813 | 8.20E-23 | G | A | 2.03E-04 | 95.50893 |
|  | rs116936375 | 10 | 18737135 | -0.02932 | 0.005404 | 2.50E-08 | A | G | 6.27E-05 | 29.44403 |
|  | rs12258967 | 10 | 18727959 | -0.03988 | 0.002235 | 1.30E-73 | G | C | 6.77E-04 | 318.4312 |
|  | rs12416030 | 10 | 18789075 | 0.016699 | 0.002578 | 3.60E-11 | C | T | 8.93E-05 | 41.95291 |
|  | rs12765864 | 10 | 18778071 | 0.019909 | 0.002196 | 2.60E-20 | T | C | 1.75E-04 | 82.15564 |
|  | rs16916944 | 10 | 18481044 | 0.022808 | 0.003001 | 5.00E-15 | T | C | 1.23E-04 | 57.74667 |
|  | rs1757216 | 10 | 18506426 | -0.02369 | 0.002889 | 1.00E-15 | A | G | 1.43E-04 | 67.24102 |
|  | rs17610275 | 10 | 18621630 | -0.02797 | 0.004045 | 3.40E-12 | G | T | 1.02E-04 | 47.8271 |
|  | rs17662793 | 10 | 18465479 | -0.01458 | 0.002253 | 1.20E-11 | G | A | 8.91E-05 | 41.86482 |
|  | rs1779209 | 10 | 18514561 | -0.02196 | 0.002275 | 3.50E-22 | C | T | 1.98E-04 | 93.14752 |
|  | rs1891392 | 10 | 18336421 | -0.01798 | 0.002243 | 5.10E-16 | C | T | 1.37E-04 | 64.2722 |
|  | rs1998822 | 10 | 18755664 | 0.01279 | 0.002303 | 3.60E-08 | G | A | 6.57E-05 | 30.84402 |
|  | rs2488152 | 10 | 18355456 | 0.022776 | 0.003366 | 2.20E-12 | G | A | 9.75E-05 | 45.7993 |
|  | rs34606998 | 10 | 18430855 | 0.015901 | 0.002407 | 1.40E-11 | T | C | 9.29E-05 | 43.65591 |
|  | rs4628581 | 10 | 18799890 | 0.011858 | 0.002062 | 5.30E-09 | C | A | 7.04E-05 | 33.0724 |
|  | rs4748457 | 10 | 18672145 | 0.019923 | 0.002965 | 5.40E-12 | G | T | 9.61E-05 | 45.14991 |
|  | rs4748508 | 10 | 18958646 | 0.014357 | 0.00205 | 3.40E-13 | G | A | 1.04E-04 | 49.03134 |
|  | rs61278674 | 10 | 18481737 | 0.0258 | 0.003536 | 1.00E-13 | G | A | 1.13E-04 | 53.23497 |
|  | rs61844175 | 10 | 18455868 | 0.019935 | 0.002774 | 3.60E-13 | A | G | 1.10E-04 | 51.63235 |
|  | rs67214975 | 10 | 18727251 | -0.02804 | 0.002055 | 1.50E-42 | A | C | 3.96E-04 | 186.175 |
|  | rs7074171 | 10 | 18676897 | 0.019324 | 0.002107 | 2.90E-20 | G | A | 1.79E-04 | 84.07295 |
|  | rs7076247 | 10 | 18759629 | -0.01685 | 0.002105 | 5.20E-16 | C | T | 1.36E-04 | 64.08062 |
|  | rs7077127 | 10 | 18717348 | 0.013364 | 0.002271 | 9.40E-10 | T | G | 7.37E-05 | 34.6211 |
|  | rs72780048 | 10 | 18340409 | -0.01096 | 0.002099 | 4.70E-08 | A | G | 5.80E-05 | 27.2656 |
|  | rs72786085 | 10 | 18713206 | -0.03301 | 0.003734 | 1.30E-18 | C | G | 1.66E-04 | 78.15167 |
|  | rs78188412 | 10 | 18708415 | -0.04026 | 0.005975 | 5.00E-12 | A | G | 9.66E-05 | 45.39742 |
|  | rs78607408 | 10 | 18386117 | -0.02772 | 0.004781 | 4.20E-09 | C | T | 7.16E-05 | 33.61465 |
|  | rs7894090 | 10 | 18291288 | -0.02004 | 0.0029 | 1.20E-11 | A | G | 1.02E-04 | 47.75671 |
|  | rs7904365 | 10 | 18831304 | 0.014207 | 0.002477 | 9.40E-09 | T | A | 7.00E-05 | 32.90489 |
|  | rs7912811 | 10 | 18379288 | -0.01259 | 0.002242 | 6.30E-09 | G | A | 6.71E-05 | 31.53472 |
|  | rs7917532 | 10 | 18373902 | -0.01531 | 0.002054 | 1.60E-14 | C | T | 1.18E-04 | 55.56356 |
|  | rs7923191 | 10 | 18727901 | 0.022118 | 0.002557 | 2.00E-18 | G | A | 1.59E-04 | 74.80968 |
|  | rs982003 | 10 | 18707296 | -0.01814 | 0.002387 | 1.40E-14 | T | C | 1.23E-04 | 57.73414 |
| CACNB3 | rs1126930 | 12 | 49399132 | 0.037861 | 0.005539 | 3.00E-12 | C | G | 9.94E-05 | 46.71347 |
|  | rs150857355 | 12 | 49209340 | 0.041681 | 0.00711 | 9.00E-10 | C | G | 7.31E-05 | 34.36232 |

Abbreviations: Chr, chromosome; SE, standard error; SNPs, single nucleotide polymorphisms; R^2^, proportion of variation explained; F, F statistics.

Table S6. Details of SNPs corresponding to drug target genes (ebi-a-GCST90018972)

| Gene | SNPs | chr | Position | Beta | SE | *P* value | Effect alleles | Other alleles | R^2^ | F |
| --- | --- | --- | --- | --- | --- | --- | --- | --- | --- | --- |
| CACNA1D | rs11130373 | 3 | 53657054 | 0.0107 | 0.0019 | 2.12E-08 | G | C | 9.32E-05 | 31.71449 |
|  | rs113210396 | 3 | 53612327 | -0.0302 | 0.0054 | 1.98E-08 | T | G | 9.19E-05 | 31.27691 |
|  | rs1547951 | 3 | 53568269 | 0.0133 | 0.0021 | 1.61E-10 | C | A | 1.18E-04 | 40.11088 |
|  | rs312487 | 3 | 53545622 | -0.0132 | 0.0019 | 2.86E-12 | C | T | 1.42E-04 | 48.26564 |
|  | rs3774620 | 3 | 53885799 | 0.0129 | 0.002 | 5.86E-11 | A | G | 1.22E-04 | 41.60226 |
|  | rs3821843 | 3 | 53558012 | 0.0186 | 0.002 | 9.33E-21 | A | G | 2.54E-04 | 86.48949 |
|  | rs62250902 | 3 | 53842251 | -0.0222 | 0.004 | 3.39E-08 | T | C | 9.05E-05 | 30.80232 |
|  | rs62250937 | 3 | 53870318 | -0.0209 | 0.0033 | 2.87E-10 | C | T | 1.18E-04 | 40.11088 |
| CACNB2 | rs10828452 | 10 | 18592450 | -0.0159 | 0.0028 | 1.86E-08 | T | A | 9.48E-05 | 32.24598 |
|  | rs11013938 | 10 | 18669271 | -0.0143 | 0.0025 | 1.85E-08 | C | G | 9.62E-05 | 32.71821 |
|  | rs11014021 | 10 | 18682233 | -0.0161 | 0.0022 | 2.18E-13 | A | T | 1.57E-04 | 53.55547 |
|  | rs112884804 | 10 | 18726977 | -0.0251 | 0.0041 | 1.08E-09 | T | C | 1.10E-04 | 37.47807 |
|  | rs12258967 | 10 | 18727959 | -0.0312 | 0.0024 | 9.88E-38 | G | C | 4.97E-04 | 168.999 |
|  | rs16916922 | 10 | 18467744 | 0.0185 | 0.0033 | 1.43E-08 | T | A | 9.24E-05 | 31.42773 |
|  | rs2357786 | 10 | 18355749 | -0.0124 | 0.0019 | 5.16E-11 | C | T | 1.25E-04 | 42.59255 |
|  | rs2497793 | 10 | 18342172 | -0.0128 | 0.0022 | 3.51E-09 | T | C | 9.95E-05 | 33.85104 |
|  | rs61841999 | 10 | 18725848 | 0.0157 | 0.002 | 1.45E-14 | A | G | 1.81E-04 | 61.62214 |
|  | rs67214975 | 10 | 18727251 | -0.0191 | 0.0019 | 7.53E-23 | A | C | 2.97E-04 | 101.0548 |
|  | rs7074171 | 10 | 18676897 | 0.0124 | 0.0021 | 6.72E-09 | G | A | 1.02E-04 | 34.86601 |
|  | rs7076319 | 10 | 18459450 | 0.0125 | 0.0021 | 5.69E-09 | G | A | 1.04E-04 | 35.43063 |
|  | rs7911644 | 10 | 18468589 | 0.0135 | 0.002 | 5.74E-12 | T | C | 1.34E-04 | 45.56223 |
|  | rs7915409 | 10 | 18419595 | 0.0302 | 0.0046 | 7.89E-11 | A | G | 1.27E-04 | 43.10183 |
|  | rs7918142 | 10 | 18755840 | -0.0118 | 0.0019 | 5.10E-10 | G | T | 1.13E-04 | 38.57041 |
| CACNB3 | rs76573653 | 12 | 49380073 | 0.0316 | 0.0058 | 4.34E-08 | G | T | 8.73E-05 | 29.68354 |

Abbreviations: Chr, chromosome; SE, standard error; SNPs, single nucleotide polymorphisms; R^2^, proportion of variation explained; F, F statistics.
